# Identifying Major Factors for Success and Failure of Conservation Programs in Europe

**DOI:** 10.1007/s00267-024-02086-x

**Published:** 2024-11-23

**Authors:** Nina Farwig, Philipp P. Sprenger, Bruno Baur, Katrin Böhning-Gaese, Angelika Brandt, Nico Eisenhauer, Götz Ellwanger, Axel Hochkirch, Alexandros A. Karamanlidis, Marion Mehring, Martin Pusch, Finn Rehling, Nike Sommerwerk, Theresa Spatz, Jens-Christian Svenning, Sabine Tischew, Klement Tockner, Teja Tscharntke, Alice B. M. Vadrot, Julian Taffner, Christine Fürst, Sonja C. Jähnig, Volker Mosbrugger

**Affiliations:** 1 Department of Conservation Ecology, University of Marburg, Karl-von-Frisch-Straße 8, 35032 Marburg, Germany; 2 Central Coordination Office of the BMBF-Research Initiative for the Conservation of Biodiversity (FEdA), Senckenberg – Leibniz Institution for Biodiversity and Earth System Research, Senckenberganlage 25, 60325 Frankfurt am Main, Germany; 3 Department of Environmental Sciences, University of Basel, Bernoullistrasse 30, 4056 Basel, Switzerland; 4 Senckenberg Biodiversity and Climate Research Centre, Senckenberganlage 25, 60325 Frankfurt am Main, Germany; 5 Department of Biological Sciences, Goethe University Frankfurt, Max-von-Laue-Straße 9, 60438 Frankfurt am Main, Germany; 6 Helmholtz Centre for Environmental Research - UFZ, Permoserstraße 15, 04318 Leipzig, Germany; 7 Senckenberg – Leibniz Institution for Biodiversity and Earth System Research, Senckenberganlage 25, 60325 Frankfurt am Main, Germany; 8 German Centre for Integrative Biodiversity Research (iDiv) Halle-Jena-Leipzig, Puschstraße 4, 04103 Leipzig, Germany; 9 Institute of Biology, Leipzig University, Puschstraße 4, 04103 Leipzig, Germany; 10 Federal Agency for Nature Conservation, Konstantinstraße 110, 53179 Bonn, Germany; 11 Musée National d’Histoire Naturelle, 25, rue Münster, L-2160 Luxembourg, Luxembourg; 12 Department of Biogeography, Trier University, Universitätsring 15, 54296 Trier, Germany; 13 ARCTUROS, Civil Society for the Protection and Management of Wildlife and the Natural Environment, Florina, 53075 Aetos Greece; 14 Faculty of Environmental Sciences and Natural Resource Management, Norwegian University of Life Sciences, 1432 Ås, Norway; 15 Institute for Social-Ecological Research, Hamburger Allee 45, 60486 Frankfurt am Main, Germany; 16 Leibniz Institute of Freshwater Ecology and Inland Fisheries, Müggelseedamm 310, 12587 Berlin, Germany; 17 Department of Animal Ecology, University of Marburg, Karl-von-Frisch-Straße 8, 35032 Marburg, Germany; 18 Chair of Nature Conservation and Landscape Ecology, University of Freiburg, Stefan-Meier-Str. 76, 79104 Freiburg, Germany; 19 Museum für Naturkunde – Leibniz Institute for Evolution and Biodiversity Science (MfN), Invalidenstraße 43, 10115 Berlin, Germany; 20 Center for Ecological Dynamics in a Novel Biosphere (ECONOVO), Department of Biology, Aarhus University, Ny Munkegade 114, DK-8000 Aarhus C, Denmark; 21 Department of Agriculture, Ecotrophology and Landscape Development, Anhalt University of Applied Sciences, Strenzfelder Allee 28, 06406 Bernburg, Germany; 22 Functional Agrobiodiversity and Agroecology, University of Göttingen, Grisebachstraße 6, 37077 Göttingen, Germany; 23 Department of Political Science, University of Vienna, Kolingasse 14-16, 1090 Vienna, Austria; 24 Department Sustainable Landscape Development, Institute for Geosciences and Geography, Martin-Luther University Halle-Wittenberg, Von-Seckendorff-Platz 4, 06120 Halle, Germany; 25 Geography Department, Humboldt-Universität zu Berlin, Unter den Linden 6, 10099 Berlin, Germany

**Keywords:** Biodiversity loss, Environmental policy, Natura 2000, Participative conservation, Rewilding, Water Framework Directive

## Abstract

In Europe, various conservation programs adopted to maintain or restore biodiversity have experienced differing levels of success. However, a synthesis about major factors for success of biodiversity-related conservation programs across ecosystems and national boundaries, such as incentives, subsidies, enforcement, participation, or spatial context, is missing. Using a balanced scorecard survey among experts, we analyzed and compared factors contributing to success or failure of three different conservation programs: two government programs (Natura 2000 and the ecological measures of the Water Framework Directive) and one conservation program of a non-governmental organization (NGO; Rewilding Europe), all focusing on habitat and species conservation. The experts perceived the NGO program as more successful in achieving biodiversity-related aims than governmental conservation legislation. Among the factors perceived to influence the success of biodiversity conservation, several stood out: Biodiversity-damaging subsidies, external economic interests competing with conservation goals or policies conflicting with biodiversity conservation were recognized as major factors for the lack of conservation success. Outreach to raise societal interest and awareness as well as stakeholder involvement were perceived as closely related to the success of programs. Our expert survey demonstrated that external factors from economy and policy often hinder success of conservation programs, while societal and environmental factors rather contribute to it. This study implies that conservation programs should be designed to be as inclusive as possible and provides a basis for developing a standardized methodology that explicitly considers indirect drivers from areas such as economy, policy and society.

## Introduction

Numerous strategies, programs and policies are directly targeted at or relevant for biodiversity conservation. These include international treaties (e.g. Convention on Biological Diversity (CBD), Convention on International Trade in Endangered Species of Wild Fauna and Flora (CITES), Ramsar Convention, Alpine Convention, Sustainable Development Goals (SDGs)), European directives and regulations (e.g. the Common Agricultural Policy (CAP), Water Framework Directive, Birds and Habitats Directives, Marine Strategy Directive, EU Biodiversity Strategy including the Nature Restoration Law) and corresponding national and sub-national laws, declarations, programs and policies (e.g. Federal Water Act or Federal Nature Conservation Act (in Germany), National Biodiversity Strategies). Measures of these strategies, programs and policies follow different conservation concepts (see Büscher and Fletcher 2020) and include (i) the protection of areas, habitats and species, (ii) active support of biodiversity (e.g. less intensive land- and sea use, environmental schemes, promotion of green infrastructure, restoration of habitats and ecosystems, reduction of introduction of non-native or invasive species, conservation action for threatened species), as well as (iii) using biodiversity sustainably (e.g. related to fisheries, forestry). Despite major efforts, the ongoing loss of biodiversity at global, regional and local levels and its various consequences for ecosystem functions, nature’s contribution to people (NCP) and human well-being, has hardly been mitigated. Indeed, despite some progress, most global and European biodiversity targets have been missed or only partly achieved (IPBES 2018, 2019; European Environment Agency 2020, 2024; Secretariat of the Convention on Biological Diversity 2020; Pörtner et al. 2021; Biermann et al. 2022; Perino et al. 2022).

Hence, the question arises: Why have conservation strategies, programs and policies around the world largely failed to halt biodiversity loss? As pointed out by the IPBES global assessment (IPBES 2019), economic, political and social factors impact nature and its contributions to people through unsustainable use of resources. Effective management and use of natural resources depend on governance and the related economic, political and social settings (Ostrom 2007). How we decide to manage our natural resources certainly has an impact on the success of conservation programs. Differing interests among and within interest groups in economy, politics and society may result in inadequate policies and regulations, trade-offs in favor of various economic activities (e.g. agriculture, forestry, infrastructure, construction, fisheries, mining), imperfect operational goals, limited coordination among programs, lack of public acceptance, as well as insufficient funding and implementation across all levels of society (Hagerman and Pelai 2016; Marselle et al. 2021; Gjerde et al. 2022). Particularly, the separate consideration of direct drivers (such as land-/sea-use change, direct exploitation, climate change, pollution, invasive species) and indirect drivers (e.g. demographic and sociocultural shifts, economic and technological drivers, institutions and governance, conflicts and epidemics) has impaired achieving global conservation targets (Pisupati and Prip 2015; IPBES 2019). While many existing programs aim to conserve selected species or ecosystems in protected areas, there are substantial taxonomic, geographic, esthetic and size biases – for instance towards charismatic species (Berti et al. 2020) – in protection status and funding allocation (Cardoso 2012; Adamo et al. 2022) that can hamper overall conservation success. Also, spatial development tools are rarely implemented sustainably on large spatial scales (but see August et al. 2002; Guerra et al. 2021; Zeiss et al. 2022). Although often legally binding, many conservation programs are implemented too slowly or inadequately, e.g. due to insufficient funding (Mulder et al. 2021; Turnhout et al. 2021). Also, lack of enforcement, limited acceptance and absence of responsibility across different stakeholders (e.g. economic actors and civil society), missing science-policy-society interfaces and/or gaps in scientific knowledge are hindering the implementation of conservation programs (Tanguay et al. 2021; Levin 2022; Sutherland 2022). Finally, current conservation programs lack adequate monitoring as well as evaluation of their success and that of their implemented measures (IPBES 2016; Guerra et al. 2021; Tessnow-von Wysocki and Vadrot 2022). In the few cases in which monitoring is mandatory, the analysis of causes for biodiversity deterioration mostly focuses on direct drivers, such as land-use change or pollution, while underlying indirect drivers (see IPBES 2019) are rarely addressed. Consequently, management plans and conservation priorities are currently inadequate, i.e. inflexible, focus too often on small-scales and are too slow to sufficiently address the ongoing challenge of biodiversity loss and thereby failing to adapt to rapidly changing environmental and social systems (Hochkirch et al. 2013).

Successful conservation programs have been shown to emerge from personal motivation, led by moral and intrinsic values, with many stakeholders considering the stewardship of nature and its preservation for future generations as a moral and intrinsic task (Admiraal et al. 2017). Moreover, appropriate governance including the enforcement of legal protection and provision of sufficient and long-term funding (measures and staff) is considered key for conservation programs (Chape et al. 2005; Black et al. 2011; Watson et al. 2014) Here, participatory processes, proactive and inclusive policies, autonomous conservation action and trust among stakeholders are essential elements (Black et al. 2011; Phillis et al. 2013; Admiraal et al. 2017; Salvatori et al. 2020; Read and Wainger 2022). For instance, active landowner participation and co-management can reduce social conflict and may increase the effectiveness of conservation programs (Noah and Zhang 2001; Young et al. 2013; Blondet et al. 2017).

As social, political, economic and environmental factors can all determine the success or failure of specific conservation programs, the comparison of existing programs can help to shed light on the role of the complex interplay of these factors. To do so, we selected two governmental conservation programs in cultural landscapes of Europe: “Natura 2000” as a protected area network and a national implementation of the Birds Directive (latest amended version Directive 2009/147/EC) and Habitats Directive (Directive 92/43/EEC) and the ecological measures of the “Water Framework Directive” (Directive 2000/60/EC) as a program with transnational management approaches systematically addressing a particular resource (in this case water). In addition, we chose an NGO-based program, “Rewilding Europe” that covers carefully chosen areas and focuses on habitats and keystone species *sensu lato* (see Table 1). Due to differences in duration, scale, target areas and conservation priorities, these programs offer different opportunities and challenges for implementation (Table 2). Nevertheless, we aimed to synthesize success and failure factors across these different conservation programs since they are all often assumed to benefit biodiversity and are grouped together in the political sphere.

**Table 1:** Features of the three conservation programs that were evaluated by experts for this study

	Natura 2000	Water Framework Directive	Rewilding Europe
**Starting Year**	1992 ^1^ (with adoption of the Habitats Directive)	2000 ^2^	2010/2011 ^3,4^
**Targets and areas**	species and habitats ^1^ almost 28,000 sites (SACs ^1^ and SPIs ^5^) across Europe, covering ~20% of terrestrial and ~10% of sea area ^7^	surface waters^2,a^ all European surface waters (at catchment scale)	restoration of ecosystem processes currently areas in ten different landscapes across Europe involving a total of 65,000 km^2^ of land and water ^4,b^
**Aims**	maintain or restore favorable conservation status of species and habitats of EU conservation interest (listed in the Annexes I and II of the Habitats Directive ^1^ and Annex I and relevant migratory bird species of the Birds Directive ^5,6^)	reach a ‘good ecological status’ in surface waters by 2027 ^2^	rewild land and create wilderness areas to complement classical nature conservation and create opportunities for nature-based economies ^3^
**Status quo (most recent assessments)**	81% of habitats and 63% of species at EU level are in bad or poor conservation status ^7^, measuring effectiveness of Natura 2000 itself is limited due to lack of appropriate monitoring ^7^	only 37% of Europe’s surface water bodies achieve a good or high ecological status yet ^8^, most protected aquatic habitats in the EU have a poor or bad conservation status ^8^	five of seven landscapes investigated improved overall performance across 19 parameters of human forcing and ecological integrity in the areas ^9^
**References**	^1^ European Commission (1992) - Council Directive 92/43/ECC ^2^ Hering et al. (2010) ^3^ Helmer et al. (2015) ^4^ Allen et al. (2022) ^5^ European Community (1979) - Council Directive 79/409/EEC ^6^ European Commission (2009) - Directive 2009/147/EC ^7^ European Environment Agency (2020) ^8^ European Environment Agency (2024) ^9^ Segar et al. (2021)		

^a^The Water Framework Directive targets all water bodies, however groundwater is only evaluated in amount and chemical quality, not in ecological status

^b^Greater Côa Valley (Portugal) since 2011, Southern Carpathians (Romania) since 2011, Velebit Mountains (Croatia) since 2012, Central Apennines (Italy) since 2013, Danube Delta (Romania, Ukraine, Moldavia) since 2013, Rhodope Mountains (Greece, Bulgaria) since 2014, Oder Delta (Germany, Poland) since 2015, Swedish Lapland (Sweden) since 2015, Affric Highlands (Scotland) since 2021, Iberian Highlands (Spain) since October 2022

**Table 2:** Opportunities and challenges of the three different conservation programs

Program	Opportunities	Challenges
**Natura 2000**	• large Europe-wide protected area network setting similar standards ^1,2^ • extension to non-targeted species possible, so far only protection of specifically targeted species and habitats ^3^	• large variety of (historical) cultural heritage and ecological context ^4^ • taxonomic and habitat bias in the allocation of funding ^5^ • taxonomic and habitat bias in the annexes of the Habitats Directive ^6,7^ as well as lack of flexibility of the species lists ^8^ • governance issues, lack of political will and overcoming negative attitudes of stakeholders ^2,9^ • way of implementation in certain countries, e.g. Central Eastern Europe ^9,10^
**Water Framework Directive**	• management on whole catchment scale allows holistic approaches in river systems ^11^ • potential to gather high quality monitoring data ^11,12^	• cross-border cooperation across different political areas ^13^ • difficult to implement due to conflicts with local stakeholders ^14^
**Rewilding Europe**	• cross-sectoral co-benefits addressed from the start ^15,16^ • high motivation of practitioners and stakeholders ^17^ • new sustainable economy-opportunities such as ecotourism etc. ^15,16^	• upscaling of the rewilding approach ^17^ • expansion of comparably small areas at selected well-suited sites ^15^
**References**	^1^ European Commission (1992) ^2^ Kati et al. (2015) ^3^ Pellissier et al. (2020) ^4^ Campagnaro et al. (2019) ^5^ Adamo et al. (2022) ^6^ Cardoso (2012) ^7^ Mammola et al. (2020) ^8^ Hochkirch et al. (2013) ^9^ Yakusheva (2019)	^10^ Mammides and Kirkos (2020) ^11^ Hering et al. (2010) ^12^ Seidel et al. (2022) ^13^ BMUB/UBA (2016) ^14^ Carvalho et al. (2019) ^15^ Helmer et al. (2015) ^16^ Jepson et al. (2018) ^17^ Allen et al. (2022)

We developed a balanced scorecard for evaluating success and failure factors for biodiversity conservation. Balanced scorecards are strategic instruments originally used in business management, using performance indicators to compare different perspectives on complex strategic activities of an organization (Kaplan and Norton 1992). We used this approach in an expert evaluation on major factors that influence success and failure of conservation programs from four principal areas – economy, society, policy and environment – and compared commonalities and differences between the conservation programs.

We formulated two research questions: (1) Is the perception of success higher in the NGO than in the governmental programs, and if yes, which role do economic, political or social factors, such as active stakeholder participation, public awareness or economic incentives play? (2) Is the perception of success higher for programs which integrate factors of several of the four above-mentioned principal areas (economy, society, policy and environment)?

We identify major influence factors and discuss promising strategies to increase the probability of success of conservation programs with the aim of fostering the integration of these four areas to enable more effective implementation.

## Methods

### Balanced Scorecard

We adapted the balanced scorecard instrument, originally used in business management, to analyze conservation programs and factors relevant to their success or failure (Fürst et al. 2014; Spyra et al. 2019). To identify relevant factors, we organized a workshop with 30 international experts (13 female and 17 male researchers from eight different countries) from diverse disciplines, such as ecology and conservation, landscape development, economics, as well as social and political sciences who were identified by the organizers through scientific networks, their previous work in biodiversity conservation and/or personal contacts as well as through suggestions of colleagues. After this initial workshop, we identified and agreed upon four principal areas relevant for biodiversity conservation (economy, society, policy and environment) and collected four success- and four failure-associated factors per area from the discussions in the workshop (32 items in total; Table 3).

**Table 3:** Failure and success factors and their definitions

Factor	Definition
*Economy*	
F1. Subsidies damaging biodiversity	Are there subsidies particularly harming the targets of the program? Examples could be subsidies promoting agricultural intensification, renewable energy production, infrastructure projects in natural areas etc.
F2. Economic interests competing with conservation	Do economic interests interfere with the conservation goals of the program? E.g. agricultural / forestry production or fisheries vs. nature conservation.
F3. Lack of funding	Is the funding of the program insufficient to successfully reach its goals? Is there not enough personnel / no financial resources for proper implementation of conservation measures?
F4. Unsustainable use of resources	Are resources of areas targeted by the programs used unsustainably and is this interfering with the programs goals / harming biodiversity?
S1. Incentives and subsidies	Do particular incentives or subsidies enhance the success of the program? Examples could be incentives for agri-environmental schemes, pollution reduction etc. Is it clear which institutions can provide incentives?
S2. Strict production standards / supply regulations	Are there strict production standards and supply regulations in regions targeted by the conservation program? Is the production of crops / timber etc. sustainable?
S3. Certification of production	Can products produced in areas targeted by the conservation program be certified as biodiversity-friendly? E.g. ecological farming, certificates for sustainable forestry / fishing etc.
S4. Promoting livelihood	Does the program promote livelihood of people in the area targeted? E.g. are there profitable ecosystem services, jobs created, ecotourism enhancing people’s livelihood?
*Society*	
F1. Conflicts of interest / ownership	Are there conflicting interests between stakeholders / landowners and the conservation goals hindering successful implementation of the program?
F2. Lack of understanding for needed transformation	Do people / stakeholders lack understanding why their behaviors interfere with conservation goals? Is there awareness that a transformation is needed?
F3. Bureaucracy and regulations	Is there unnecessary bureaucracy and regulations that hinder the program’s successful implementation? Are there regulations that allow sanctions for imperfect measure implementation?
F4. Underestimating problem of biodiversity loss	Are people underestimating the problem of biodiversity loss leading to limited acceptance of the program? Is biodiversity loss recognized in local communities?
S1. Biodiversity awareness	Became people more aware of biodiversity and the benefits provided e.g. through ecosystem services? Does this enhance acceptance of the program leading to more successful implementation?
S2. Positive outreach, raise interest, grassroot initiatives	Are people interested in the program? Are there success stories for a positive outreach promoting grassroot initiatives to support the program? Are NGOs or volunteers engaging in the program?
S3. Local acceptance / Collective decision-making	Is the program locally accepted? Are local people involved in decision-making and is this promoting long-term success of the program? Is the program respecting values and interests of local stakeholders?
S4. Education / Capacity building	Does the program help in education and capacity building for enhancing the long-term success? Does the program provide opportunities for environmental education, e.g. for school or university students?
*Policy*	
F1. Policies damaging biodiversity / Conflicting policies	Are there conflicting policies hindering the successful implementation of the program? E.g. Biodiversity Strategy and Renewable Energy Directive. Do other policies harm biodiversity in the area targeted by the program?
F2. Lack of science-policy interfaces and counseling	Are decision makers insufficiently informed about scientific evidence hindering effective policies? Is there insufficient counseling for decision makers hindering effective program implementation?
F3. Lack of awareness	Are decision makers not aware enough about conservation issues hindering effectiveness of policies?
F4. Lack of action / priorities	Is there a lack of action in program implementation? Are there other political priorities hindering the implementation of the program?
S1. Cross-sectoral cooperation and biodiversity mainstreaming	Does cross-sectoral cooperation enhance the success of the program? Is biodiversity also considered across sectoral policies (i.e. biodiversity mainstreaming)?
S2. Establish fitting conditions through reforms	Did political reforms establish fitting conditions enhancing the success of the program? Are measures of the program easy to apply without too many conflicting interests or conflicting polices?
S3. Internalize environmental costs	Are environmental costs internalized to promote the success of the conservation program?
S4. Reliability of funding (ease of access, continuity and sufficiency)	Is there long-term continuity in funding? E.g. is it easy enough to apply for funding or incentives? Is there a risk to pay back if actions fail?
*Environment*	
F1. Lack of spatial and temporal connectivity	Are protected areas / areas of program implementation spatially and temporally connected enough? If not, is this hindering conservation success?
F2. Deterioration at other / different places (net loss of natural areas)	Is there deterioration of natural areas outside of protected areas? Is there net loss of natural area? Is the area around the area targeted by the program intensively used and has this a negative impact on the protected site?
F3. Untargeted conservation	Is conservation targeted at specific habitats or species or too untargeted to lead to successful conservation? Is the context of the conservation measures clearly focused on e.g. agricultural landscapes, river ecosystems, marine protected areas etc.?
F4. Lack of appropriate monitoring / data	Is the monitoring infrastructure and data present appropriate to implement the conservation program successfully? Is evaluation of success based on existing monitoring data?
S1. Understanding ecological context	Does the understanding of local ecological context lead to successful implementation of the program?
S2. Appropriate management in spatial context	Is the local spatial context implemented in the management plans? Are participatory approaches in management successfully implemented?
S3. Area size and quality	Is the size and quality of the area targeted by the program sufficient? If yes, does this yield in successful conservation?
S4. Presence or establishment of local species pools	Is the local species pool still rich enough (species diversity and genetic diversity) to promote population growth? Is it possible to successfully re-introduce species for successful conservation? Are re-introduced individuals genetically adapted to local conditions?

The table shows four failure (F1-F4) and four success factors (S1-S4) per area (economy, society, policy and environment) and their defining questions from the scorecard survey filled in by the experts

The balanced scorecard allows a semi-quantitative analysis of the performance of different conservation programs. We selected three European conservation programs (in a broad sense) to reflect the diversity of governmental and non-governmental instruments. We chose two government programs on a large spatial scale: Natura 2000 and the ecological measures of the Water Framework Directive as two instruments dedicated to different ecosystems. Complementarily, we chose Rewilding Europe as an NGO-based program focusing on a wide range of different ecosystems (note the different runtime of the programs, see Table 1).

To assess these three programs, we reached out to 86 national and international experts from academia, NGOs and governmental institutions via e-mail that are renowned for their expertise in the development and implementation of these specific conservation programs (i.e. through reports and scientific publications on these specific conservation programs and through personal contacts of the authors). Care was taken to find an equal number of experts per program and an equal number of women and men. The selected experts have a strong background in various aspects of conservation, such as scientific overview of European conservation programs, evaluating the effectiveness of specific programs and carrying out practical conservation measures. We requested them to complete the balanced scorecard survey (Table S1) against this expertise as part of the evaluation process and send it back to us via e-mail. In total, 28 of those experts completed scorecards for one or more programs (32.5% response rate; 12 for Natura 2000, 10 for the WFD, 8 for Rewilding Europe). Of those, 15 male and 13 female experts from seven, mostly Central European, countries completed the survey (68% of them from Germany; see Fig. S1 for detailed information on gender, nationality and profession of the participating experts). The response rate of 32.5% for our study is comparable with the average response rate in meta studies on web surveys (e.g. Daikeler et al. 2022) and another Delphi survey with conservation experts (Curzon and Kontoleon 2016). Despite the geographical bias in our list of experts, which should be considered when interpreting the results, they still have expertise in the analyzed conservation programs and their implementation across a wide range of Europe.

The factors (technically ‘items’ in our survey), were ranked on a Likert scale from 0 to 5 (success) and −5 to 0 (failure) by the experts. To clarify definitions of the factors, we formulated standardized questions for the evaluation of factors and included these in the scorecard table (Table 3 and S1). To assess uncertainty related to these perceptions, the respondents were also asked to disclose how confident they were regarding their responses (A—very confident; B—intermediate; C—unsure; Table S1).

Following the initial response, the results of the group were synthesized and sent back to the participants, who were then asked to either confirm or correct their votes (1-stage Delphi approach) compared to the average group votes (Dalkey and Helmer 1963; Gorn et al. 2018). All participants had replied to this request, either correcting some of their votes or confirming their initial response, without any dropouts. In the results, we present only the consolidated results from the second round of voting. All votes of phase 1 can be found in the supplement (Table S2). Comparisons of scores and uncertainties between the two phases are provided in the Supplementary Results.

### Data Analysis

As we provided interval scales for the assessments in our balanced scorecard, we were able to use parametric statistics for analysis. We analyzed the effect of conservation programs, the area (economy vs. policy vs. society vs. environment) and type (success vs. failure) of a factor and all of their interactions (up to three-way) on expert scores with linear mixed models and included the identity of experts as a random factor. For the model, we added the value 5 to the scores of failure factors to harmonize values from different Likert scales of factors associated with failure and with success (transforming the scale of −5 to 0 to 0 to 5). However, we present expert scores on their original scale throughout the text. The overall balanced score was calculated as the sum of failure and success scores per area (which are the mean of the four factors in each area; see Table S1). Confidence levels (A–C) of the expert evaluations were transformed into an uncertainty score ranging from 0 (no uncertainty) to 1 (high uncertainty) as the mean of confidence levels. For calculation, the levels were scored with A = 0 for the highest confidence level, B = 0.5 for the intermediate and C = 1 for the lowest confidence level (i.e. highest uncertainty).

**Fig. 1:**
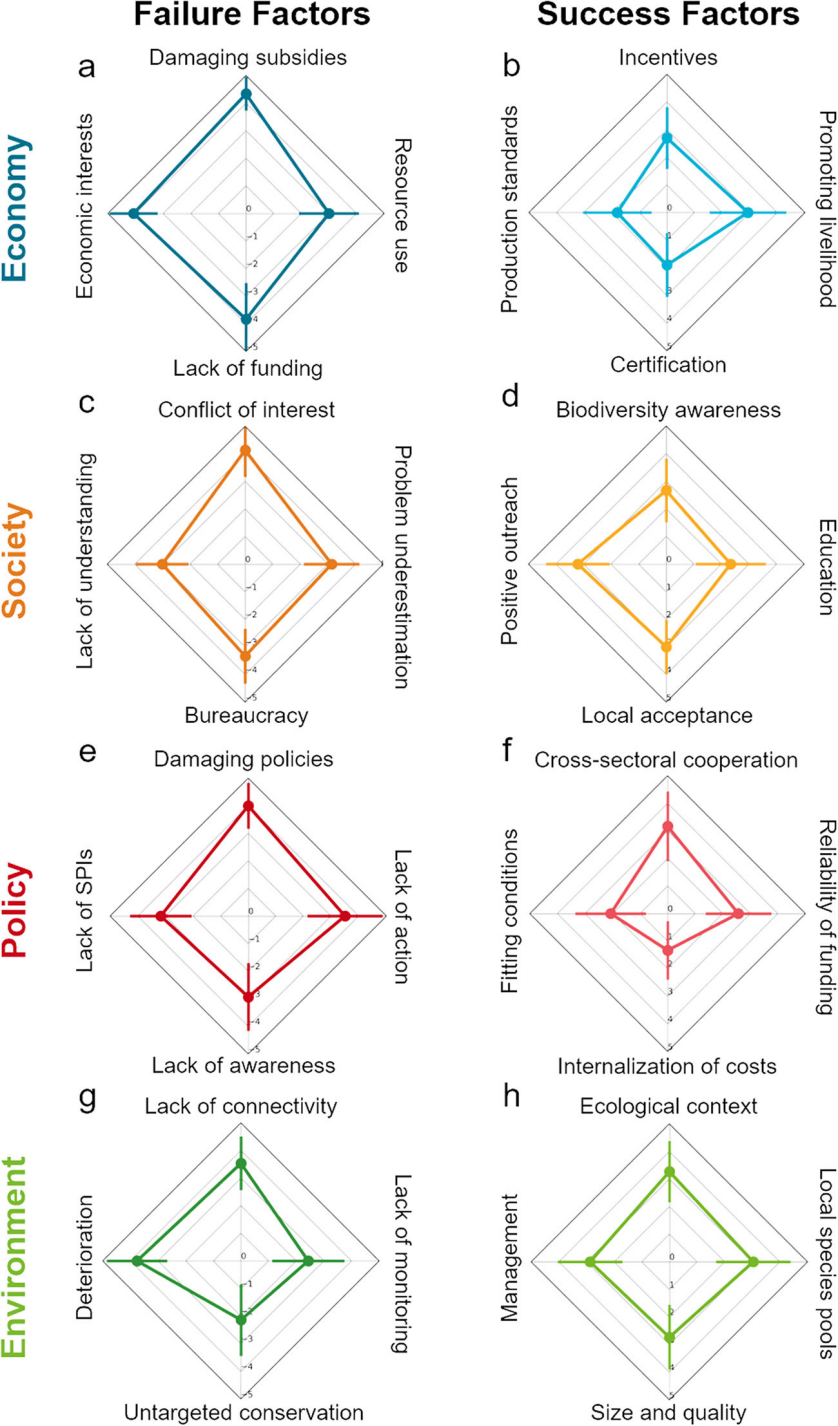
Consolidated results of expert evaluations for factors in total. Spider plots show mean factor scores (dots) and their standard deviations (error bars) of the second expert evaluation round (based on the raw data). Failure factors range from 0 (central) to −5 (marginal); success factors range from 0 (central) to 5 (marginal); **a**, **c**, **e** and **g** show results for failure factors, **b**, **d**, **f** and **h** for success factors; each in the order economy, society, policy and environment

**Fig. 2:**
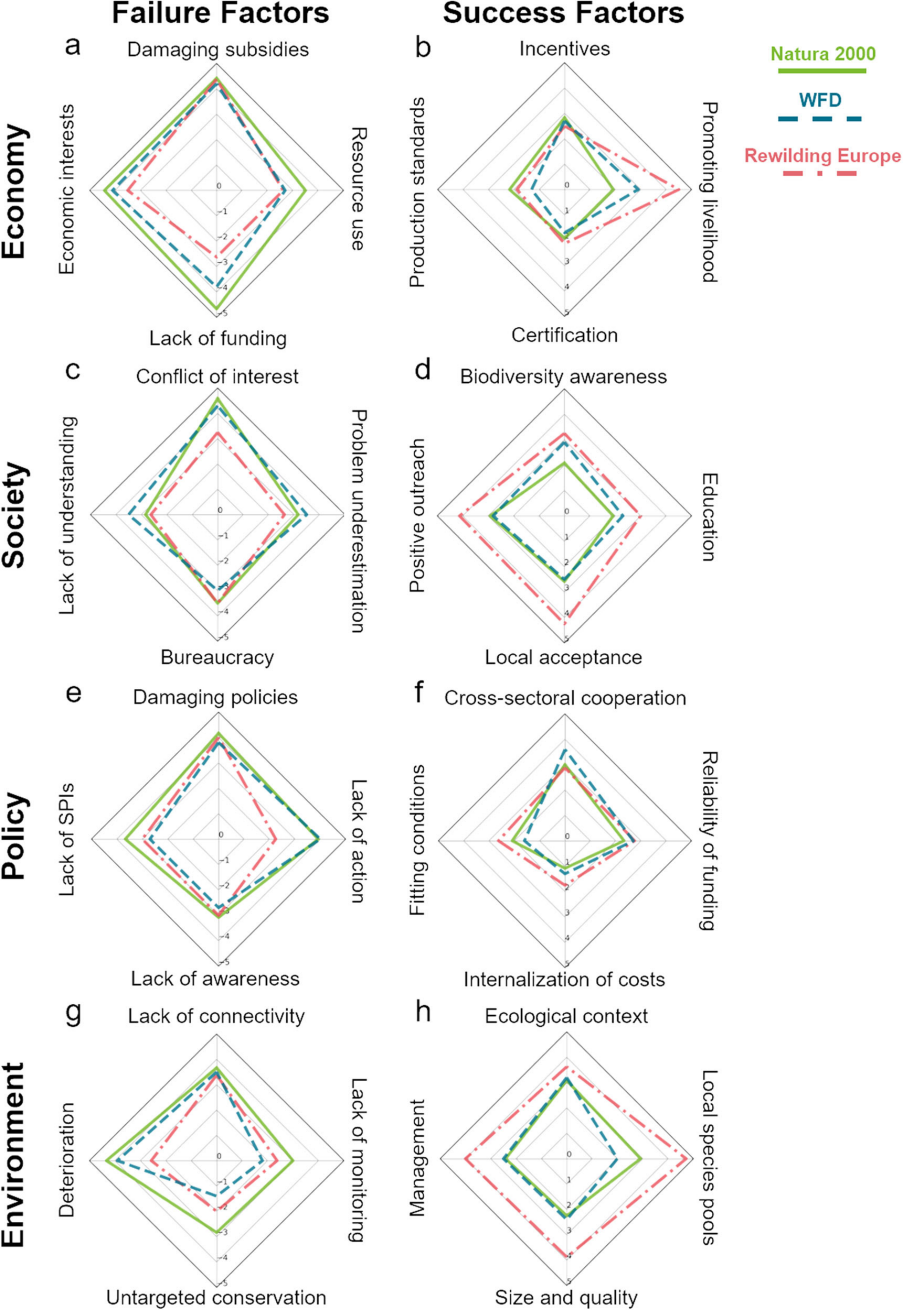
Consolidated results of expert evaluations for factors per program. Plots show mean factor scores per program with Natura 2000 in green, the Water Framework Directive in blue and Rewilding Europe in red for the second expert evaluation round (based on the raw data). Failure factors range from 0 (central) to −5 (marginal); success factors range from 0 (central) to 5 (marginal); **a**, **c**, **e** and **g** show results for failure factors, **b**, **d**, **f** and **h** for success factors; each in the order economy, society, policy and environment. For standard deviations of the scores see Table 4

All statistical analyses were performed using *R* version 4.2.2 (R Core Team 2022). Linear mixed models were constructed using the package *‘glmmTMB’* version 1.1.5 (Brooks et al. 2017). Significance values for the effect of fixed factors were obtained with Wald-χ² tests in the package *‘car’* version 3.1.1 (Fox and Weisberg 2019) and model performance was evaluated using the package *‘DHARMa’* version 0.4.6 (Hartig 2022). Spider charts (Figs. 1 and 2) were created using *Python* version 3.10.5 and *‘matplotlib’* version 3.3.0.

## Results

### Perception of Success Among Programs

The strongest effect in our analysis was that the failure factors overall were scored more strongly (more negative) on the negative scale (−5 to 0) than success factors on the positive scale (0 to 5) (GLMM: χ²_1_ = 183.88, *p* < 0.001). Expert scores of Rewilding Europe were consistently higher than those of the government programs, i.e. Natura 2000 and the WFD (χ²_2_ = 24.06, *p* < 0.001), confirming our first research question. This is evident in the overall balanced scores (Natura 2000: −5.48 ± 2.60; WFD: −3.63 ± 2.49; Rewilding Europe: 0.96 ± 3.95; Table 4).

**Table 4:** Consolidated results of the expert evaluations on conservation programs

The table shows the means and standard deviations of the expert evaluations in the second round for each failure (negative) or success factor (positive), as well as the scores for each area and overall balanced scores, in total and per program (Natura 2000, the Water Framework Directive [WFD] and Rewilding Europe). The top 4 success and failure factors per column are printed in bold (success factors in green, failure factors in red). Below these values, we show the scores of uncertainties in italics, which range from 0 (no uncertainty) to 1 (high uncertainty). Scores for Rewilding Europe marked with asterisks have *n* = 7 due to missing evaluations

Expert scores also differed among the four principal areas (χ²_3_ = 59.26, *p* < 0.001) and were generally higher for the area “environment” than for the other areas. The combined scores were most negative for the area “economy”, followed by the area “policy” throughout all three programs (Table 4). Interestingly, Rewilding Europe showed negative impacts of the areas “policy” and “economy” (economy: −0.53 ± 1.04; policy: −0.56 ± 1.53) comparable to the other two programs. While the combined scores for “society” and “environment” were also negative in total for Natura 2000 and the WFD, they were positive in Rewilding Europe (Table 4), pointing towards more positive perception of the NGO program throughout all four areas, which gives answer to our second research question (more success for programs considering all four areas simultaneously).

Our model also showed a three-way interaction between conservation programs, area and type of factor (χ²_6_ = 15.84, *p* = 0.015; all model results in Table S3), implying that success and failure factors were scored with varying strengths across the four areas and three conservation programs (see Fig. S2).

### Identification of Failure and Success Factors in the Different Areas

#### Economy

In the area “economy”, scores for failure factors were scored more strongly (more negatively) compared to other areas (Fig. S2). “Subsidies damaging biodiversity” and “Economic interests competing with conservation” had the most negative scores overall (−4.33 ± 0.61 and −4.07 ± 0.87; Fig. 1a), independent of the conservation programs and with low overall uncertainty (Fig. 2a; Table 4). Scores for economic failure factors were stronger (more negative) in Natura 2000 compared to the other programs (Fig. S2) and especially “Lack of funding” was perceived as a major negative factor for Natura 2000 (−4.67 ± 0.49; Fig. 2a). In contrast, the economic success factor “Promoting livelihood” (of people) had very positive scores for Rewilding Europe with no uncertainty (4.50 ± 0.76; Fig. 2b; Table 4).

#### Society

Success factor scores for the area “society” were often higher for Rewilding Europe (Fig. S2). Especially “Local acceptance and collective decision-making” was evaluated as a contributor to the success of Rewilding Europe (4.25 ± 0.71; Fig. 2d), while “Positive outreach, raise interest, grassroot initiatives” was perceived as an important success factor throughout all conservation programs (3.20 ± 1.16; Fig. 1d). On the other hand, “Conflict of interest / ownership” was rated as a negative influence on conservation programs (−4.13 ± 0.97; Fig. 1c; low uncertainty, see Table 4), especially in the government programs (Natura 2000: −4.58 ± 0.51; WFD: −4.30 ± 0.48; Fig. 2c). Scores of “Bureaucracy and regulations” were low for Rewilding Europe (−3.50 ± 0.93; Fig. 2c), albeit with some uncertainty of the experts (Table 4).

#### Policy

Factors of the area “policy” were generally scored low, i.e. failure factors had a strong contribution, while success factors did not (Table 4; Fig. S2). The factor “Policies damaging biodiversity / Conflicting policies” was rated negatively for all conservation programs (−4.00 ± 0.83; Figs. 1e, 2e), while “Cross-sectoral cooperation and biodiversity mainstreaming” was perceived as a factor for success in Natura 2000 (3.00 ± 1.41) and the WFD (3.60 ± 1.17; Fig. 2f), albeit with a slightly higher uncertainty in the latter (Table 4).

#### Environment

Success factors for the area “environment” were scored higher than for the other areas, especially for Rewilding Europe (Table 4; Fig. S2). “Understanding the ecological context” (3.27 ± 1.11) and “Presence or establishment of local species pools” (3.03 ± 1.35) were perceived as the most important success factors throughout (Fig. 1h) and the latter was scored most positive for Rewilding Europe with less uncertainty compared to Natura 2000 (4.71 ± 0.49; Fig. 2h; Table 4). Failure factors for Natura 2000 were scored especially negative for “environment” (Fig. S2), with “Deterioration at other / different places (net loss of natural areas)” being perceived as a major contributor to conservation failure (−4.33 ± 0.65; Fig. 2g; low uncertainty, Table 4).

## Discussion

Our survey conducted across three different conservation programs in Europe consistently showed that biodiversity-damaging subsidies and conflicting economic interests were perceived as the most relevant failure factors contributing to challenges in conservation implementation. In contrast, in our Central European-dominated analysis, understanding the ecological context and positive outreach to raise societal interest, local acceptance and awareness were identified as major factors for the success of these conservation programs.

### Differences and Similarities between Natura 2000, the WFD and Rewilding Europe

Rewilding Europe, an NGO-governed conservation program that engages various stakeholders (question 1) and considers economic, societal, policy and environment factors simultaneously (question 2) was consistently perceived as more successful for biodiversity conservation than Natura 2000 and the ecological measures of the Water Framework Directive (in anthropogenic landscapes; e.g. Salvatori et al. 2020). This is likely due to a high demand of land and water resources in human-dominated landscapes, which makes it essential for conservation efforts to take the various interests and needs of different areas into account in order to be effective (Berkes 2004; Chape et al. 2005; Palomo et al. 2014). By engaging a wide range of stakeholders and considering the complex interplay between different factors and areas, such as inclusive policies, economic incentives, mutual understanding and ownership (see Fig. S2), conservation programs may be able to identify and address the root causes of conservation challenges and work towards inclusive and sustainable solutions that benefit both the environment and human communities (Cook et al. 2013; Frank and Glikman 2019). These major factors, i.e. public acceptance and promoting people’s livelihood (Fig. S2) were ranked as the main success factors for Rewilding Europe (also see Segar et al. 2021). It is important to keep in mind that this positive perception of Rewilding Europe may partly be due to the fact that it is a relatively young program covering only selected areas with good potential for successful implementation (Helmer et al. 2015), while the choice of Natura 2000 sites is mainly determined by the occurrence of the target species and habitats listed in annexes of the Birds and Habitats directives. Positive perception in large-scale government programs such as Natura 2000 is often perceived if management measures are partially implemented in a participatory way or with financial incentives (e.g. Sheail 1995; Otsus and Harak 2005). Implementation challenges of the government programs evaluated were related to failure factors in economy and policy (e.g. lack of funding and lack of action, Fig. 2). Previous work also found a lack of political will and negative attitude of societal stakeholders constraining the implementation of Natura 2000 (e.g. Kati et al. 2015; Blicharska et al. 2016). Likewise, boundaries among politics, socio-economy and the environment have been associated with challenges in the implementation of the Water Framework Directive (e.g. Birol et al. 2006; Hering et al. 2010; Moss 2012; Berbel and Expósito 2018; Carvalho et al. 2019). This indicates that conservation programs require harmonized policies for effective enforcement and implementation (e.g. Gruber et al. 2012; Hermoso et al. 2016), coupled with financial incentives and mutual understanding for biodiversity conservation (e.g. Sheail 1995; Otsus and Harak 2005). Stakeholder involvement can reduce conflicts as accentuated participation, awareness and ownership are the major factors for implementation success (e.g. Blicharska et al. 2016; Gallo et al. 2018; Salvatori et al. 2020).

Our survey confirms the need for a comprehensive set of measurable and verifiable biodiversity indicators that allow assessing the progress towards achieving objectives and the success of conservation programs. While protected areas are often seen as the cornerstone for biodiversity conservation (Langhammer et al. 2024; Riva et al. 2024), assessing their true effectiveness remains challenging (e.g. Gray et al. 2016; Visconti et al. 2019; Rodrigues and Cazalis 2020). The new Global Biodiversity Framework (GBF) will move forward in this respect by hopefully fostering integrative socio-ecological ways that consider the different needs of various stakeholders to effectively protect 30% of the planet by 2030 (e.g. Palomo et al. 2014; CBD 2022; Stokstad 2022; but note the critique on protected area approaches as center piece for biodiversity conservation, e.g. Salafsky et al. 2001; Stem et al. 2005; Chazdon et al. 2009). Moreover, the new GBF aims at restoring “at least 30 percent of areas of degraded terrestrial, inland water and coastal and marine ecosystems” and at reducing excess nutrients and hazardous chemicals by half (CBD 2022). The European Union adopted the EU Nature Restoration Law in June 2024 (Regulation 2024/1991; European Commission 2024), taking a big step towards legal implementation of this GBF target 2. Along with the reduction of harmful subsidies and the increase of incentives for biodiversity conservation (CBD 2022), these goals will hopefully foster mainstreaming of biodiversity actions more strongly across all areas and may be an effective political step to bending the curve of biodiversity loss. All of these aims and goals depend on solid and swift implementation.

### Synergies and Conflicts

Corresponding to our research questions, our assessment of factors associated with the success or failure of conservation programs (as perceived by our experts) revealed higher implementation success for Rewilding Europe. That is probably the case because it simultaneously considers co-benefits across different areas (i.e. economy, society, policy, environment) in planning and implementation (Palomo et al. 2014). For instance, the combination of specifically selected areas (ecological context), which particularly comprised the potential for positive development and management of targeted habitats and species and the economic incentives for the local community, was beneficial for Rewilding Europe (Helmer et al. 2015). As mentioned previously, such beneficial circumstances are not always present in all landscapes covered by large-scale government conservation programs such as Natura 2000 or the WFD. Rewilding Europe’s approach to restore ecological integrity (see Karr and Dudley 1981) via restoring local species pools of missing keystone species *sensu lato*, notably functionally important megafauna species (trophic rewilding; Svenning et al. 2016, 2024), may also help in management of natural areas (e.g. Natura 2000 sites) in which ecosystem functions are currently disrupted (Timmermann et al. 2015).

The responses of our survey emphasized the high potential of synergies across society and environment even stretching to economy as these areas can be linked via nature’s contribution to people, such as food security, clean water, human and ecosystem health and others (Fig. 2 and S2). Existing evidence indicates that incorporating these ecosystem services into communication, public awareness, decision-making and implementation of conservation programs is key to their success (e.g. Daily and Matson 2008; Potschin and Haines-Young 2011; Primmer et al. 2015). However, economic and political factors, such as (a) damaging subsidies, (b) conflicting economic interests and (c) bureaucracy and (unnecessary) regulations, emerged as negative factors associated with challenges in the programs of our survey. This reveals a strong divide between best-practice knowledge and economy interest-led implementation (Fig. 2 and S2). Strategies that explicitly consider co-benefits across areas and integrate them across spatial scales are necessary (e.g. Reed et al. 2020). The global target to progressively exchange subsidies that harm biodiversity for positive incentives for biodiversity conservation and sustainable use will be a step towards the provision of such synergies across the economy and the environment (CBD 2022). Hereby, the relevance of highlighting common goods and services of ecosystems has been key for promoting the assumption of personal responsibility within society (Sikor et al. 2014). This aspect was also identified as a major factor for Rewilding Europe in our survey (“Positive outreach, raise interest, grassroot initiatives”). Participatory conservation programs are known to promote cooperation across areas and thereby could reduce harmful subsidies or resolve conflicting legislations (Nel et al. 2016), as well as integrate their implementation in a coherent spatial and landscape planning system (Opdam et al. 2002). Similarly, prior studies evaluating conservation implementation attributed its success to the existence of legislation and policy, adequate economic funds and public outreach into the society (Keeley et al. 2019).

### Methodological Limitations

The use of a balanced scorecard approach to compare selected conservation programs was based on the motivation to generate a standardized assessment framework related to multiple success and failure factors in four areas, how effective these different conservation programs were perceived and what their strengths and weaknesses may be. Comparable approaches were already implemented in diverse contexts of assessing biodiversity conservation regarding the multiple use of nature (Gomes et al. 2021; Sobkowiak 2022). Limitations of our approach lay in the semi-quantitative assessment of factors as implemented through a Likert scale. To reduce this limitation, we documented uncertainties, (a) by directly asking the experts about how certain or uncertain they felt with their response (Gorn et al. 2018); and (b) by implementing a 1-stage Delphi survey, so that experts had the opportunity to correct their assessment in light of the overall group perception. Such repeated assessments allow single participants to reflect on their answers and thereby correct or maintain their perspective and thus provides indirect evidence of how similar or diverse perceptions in qualitative assessments are (see e.g. Glass et al. 2022).

One aspect in assessments is the selection of participants representing expert knowledge. In our case, the discursive process in the initial workshop already provided a sound overview of experts related to exploring the effectiveness and performance of different programs related to biodiversity conservation. The perceptions reflected here are a first attempt to approach biodiversity conservation programs and actions in a more standardized and strategic way in order to help identify potential pitfalls. As perceptions of biodiversity loss and its drivers may differ depending on gender, cultural and geographic background and interactions between those (Isbell et al. 2023), such factors would be ideally considered, which however is not possible here due to the limited number of experts participating in the survey. In our survey, it is important to note that the majority of experts was primarily from Central European countries (Fig. S1). While considering this limited perspective, we still believe that our findings possess potential applicability across various spatial levels and diverse sociocultural backgrounds, as the experts completed the balanced scorecard against their diverse expertise and backgrounds.

Another limitation was that some of the factors were highly condensed and thus covered several (sometimes controversial) aspects that might have had to be assessed differently. We have no other solution for this shortcoming than explaining and raising awareness. For instance, some of the experts claimed that for “Bureaucracy and regulations”, unnecessary bureaucracy is potentially a different issue than reasonable regulations that allow sanctioning. For “Deterioration at other / different locations (net loss of natural area)” the definitions asked not only for deterioration outside protected areas, but also whether or not intensive use outside protected areas impacts biodiversity inside. Similarly, some other factor definitions contained such controversial aspects that did not always allow generalizations. Because of such issues in the definitions, inaccuracies in valuation and increased uncertainties for these factors may have resulted.

## Perspectives

While complex, the results of our study may allow some conclusions that can help to improve the implementation and/or the transformation of existing and the development of future conservation programs.

Most importantly, we found that without strong endorsement of a conservation program by local communities and stakeholders, biodiversity conservation goals are less likely to be successfully achieved. In a complex governance landscape, there are always numerous loopholes for non-cooperative stakeholders to undermine conservation programs and cause them to fail – especially when monitoring and enforcement measures are underfunded and cannot be implemented consistently and systematically. This finding is not new and is in line with other observations that conservation programs are more successful if stakeholders have personal interests in conservation, such as moral and intrinsic motivation to preserve nature for future generations and actively participate in developing measures and management plans (Young et al. 2013; Blondet et al. 2017). When this is backed up with benefits and/or financial incentives, the likelihood of adopting conservation management on multiple scales increases (Read and Wainger 2022). Indeed, trust among stakeholders, representatives of different interest groups and the civil society fosters mutual understanding and inclusive policies across all levels of society (Cook et al. 2013; Frank and Glikman 2019).

The need for strong participatory components in biodiversity conservation is also recognized by the Conference of the Parties to the CBD (2022) and called for in several of its 23 targets. The need for full, equitable, inclusive, effective and gender-responsive participation (including Indigenous peoples and local communities, see Target 22) has far-reaching implications for establishing new conservation programs and improving existing ones. Above all, it means that design, implementation and monitoring of new conservation programs require transdisciplinary approaches that combine scientific and non-scientific “orientation-” (or “target-”), “transformation-”, “systems-” and “process-knowledge” in a complex, iterative process (Reyers et al. 2010; Jahn et al. 2012; Karrasch et al. 2022; Lawrence et al. 2022). One disadvantage of such transdisciplinary processes could be that conservation programs developed that way are not specifically optimized for biodiversity conservation, but rather represent compromises reflecting different interests (see Lawrence et al. 2022). However, our study suggests that this shortcoming is overcompensated by having the support of all stakeholders if conservation programs are designed and implemented in a transdisciplinary way. This way, such transdisciplinary conservation programs may protect biodiversity more effectively (Margules et al. 2020) than current conservation actions (Langhammer et al. 2024).

Consequently, the development, monitoring and controlling of conservation programs should no longer be a task for government agencies or biodiversity- and conservation experts alone, but rather must involve and engage various stakeholders including Indigenous peoples and local communities. Processes such as co-design, co-production and co-implementation, as well as co-dissemination and shared communication by expert and non-expert stakeholders must become key elements of existing and new conservation programs (Moser 2016; Norström et al. 2020). Arguably, participatory systemic conservation programs as described above are associated with more effort regarding the design, implementation and monitoring. Also, they are more likely to provide coherent system solutions (and not just mono-functional solutions; see above) that are accepted by a critical mass of stakeholders across all areas. However, it will not always be possible to fully adopt this idealized transdisciplinary path to develop, implement and monitor conservation programs.

Successful conservation programs should be accompanied by campaigns to create biodiversity awareness so that all people impacted by a conservation program are informed and aware of the benefits that will outweigh short-term disadvantages within an acceptable time period (see Ryan et al. 2020 and references therein). Importantly, it must be recognized that biodiversity operates according to natural ecological laws, meaning that if a program ends up adopting ecologically suboptimal actions due to preferences of stakeholders, this will lead to suboptimal effects on biodiversity. Therefore, it will often be important to convince stakeholders that it is worthwhile to accept changes or restrictions in order to promote and protect biodiversity. Although our findings show that biodiversity awareness and mainstreaming across areas strongly contribute to the success of the three conservation programs (Fig. 2 and S2), they are the most often neglected components (Perino et al. 2022; de Oliveira Caetano et al. 2023). In fact, common practice outreach measures of communication and information, are not sufficient in this context. Instead, truly participatory, transdisciplinary approaches are required for creating awareness and thus also ownership in stakeholders.

## Conclusion

In summary, while evaluating the conservation programs examined here, three major factors emerge as pivotal determinants of success or failure: the degree of stakeholder participation, potential conflicts arising from economic interests or ownership concerns and the nuanced interplay of various other influencing factors. Our study underscores the significance of these factors, akin to “Liebig’s Law of the Minimum” (von Liebig 1855), wherein the success of a conservation program may hinge upon the alleviation of the most limiting factor specific to each stakeholder group’s circumstances and perspectives. Recognizing the risk of a “minimum factor” effect, where the success of a program is constrained by its weakest component, it becomes imperative to enhance conservation efforts holistically. Correspondingly, the most effective strategy, especially in government programs, is to consider all four areas and their interrelationships to minimize conflicts while optimizing synergy effects, with a particular focus on economic factors. In view of numerous different approaches for evaluation of conservation programs, we would like to conclude by recommending the development of a standardized evaluation procedure that not only captures the success or failure of a conservation program, but also the causes of success or challenges that might have hampered success so far. Our study provides part of a basis and relevant information for developing such a standardized methodology that explicitly considers the indirect drivers from areas such as economy, policy and society.

## Supplementary information

Supplementary Information

## Data Availability

Raw data and code are available from GESIS Data Archive, Cologne, via 10.7802/2790.
