# Supplementary material for: Identifying Major Factors for Success and Failure of Conservation Programs in Europe
Source: Environ Manage. 2024 Nov 23;75(3):425–43. doi: 10.1007/s00267-024-02086-x (PMC11861224; doi:10.1007/s00267-024-02086-x)
Supplement: Supplementary file 1 — Supplementary Information [file 267_2024_2086_MOESM1_ESM.docx]

**Supplementary Material of “Identifying major factors for success and failure of conservation programs in Europe”**

**Supplementary Results:**

*Evaluation of the Delphi process*

The mean scores for each factor did not significantly differ between evaluation rounds for all programs together (paired t-test: t_31_ = 1.91, p = 0.065; Supplementary Table S2) as well as separately for Natura 2000 and Rewilding Europe (each p > 0.01). For the Water Framework Directive (WFD) however, scores were slightly lower in the second evaluation round (t_31_ = 2.19, p = 0.036).

The re-evaluation of the results in the 1-stage Delphi process significantly reduced the uncertainties in the evaluated factors (t_31_ = 4.12, p < 0.001). However, when looking at the programs separately, uncertainty decreased for Natura 2000 (t_31_ = 2.96, p = 0.006) and Rewilding Europe (t_31_ = 4.68, p < 0.001), while it slightly increased for the WFD (t_31_ = -2.67, p = 0.012). Also, standard deviations were lower in the second evaluation round, both for all programs together (t_31_ = 9.07, p < 0.001), as well as for the programs individually (each p < 0.0015).

**Supplementary Table S1: Balanced scorecard for the evaluation of nature conservation programs.** The balanced scorecard as it was handed to the experts for the evaluations. The factors were evaluated on a Likert scale 0 (not at all) to |5| (fully applies). In addition, a measure of uncertainty (confidence levels) was evaluated at three levels (A - very confident; B - intermediate; C - unsure).

|  |  |  | **Areas / Factors** |  |  |  |  |  |  | **Confidence Level** | |  | **Scores** | **Definitions** |
| --- | --- | --- | --- | --- | --- | --- | --- | --- | --- | --- | --- | --- | --- | --- |
|  | **Economy** | **Failure Factors** | 1. Subsidies damaging biodiversity | -5 | -4 | -3 | -2 | -1 | 0 | A | B | C |  | Are there subsidies particularly harming the targets of the program? Examples could be subsidies promoting agricultural intensification, renewable energy production, infrastructure projects in natural areas etc. |
|  |  |  | 2. Economic interests competing with conservation | -5 | -4 | -3 | -2 | -1 | 0 | A | B | C |  | Do economic interests interfere with the conservation goals of the program? E.g. agricultural / forestry production or fisheries vs. nature conservation. |
|  |  |  | 3. Lack of funding | -5 | -4 | -3 | -2 | -1 | 0 | A | B | C |  | Is the funding of the program insufficient to successfully reach its goals? Is there not enough personnel / no financial resources for proper implementation of conservation measures? |
|  |  |  | 4. Unsustainable use of resources | -5 | -4 | -3 | -2 | -1 | 0 | A | B | C | *Economy Failure Score: Mean value failure factors 1-4* | Are resources of areas targeted by the programs used unsustainably and is this interfering with the programs goals / harming biodiversity? |
|  |  | **Success Factors** | 1. Incentives and subsidies | 0 | 1 | 2 | 3 | 4 | 5 | A | B | C |  | Do particular incentives or subsidies enhance the success of the program? Examples could be incentives for agri-environmental schemes, pollution reduction etc. Is it clear which institutions can provide incentives? |
|  |  |  | 2. Strict production standards / supply regulations | 0 | 1 | 2 | 3 | 4 | 5 | A | B | C |  | Are there strict production standards and supply regulations in regions targeted by the conservation program? Is the production of crops / timber etc. sustainable? |
|  |  |  | 3. Certification of production | 0 | 1 | 2 | 3 | 4 | 5 | A | B | C |  | Can products produced in areas targeted by the conservation program be certified as biodiversity-friendly? E.g. ecological farming, certificates for sustainable forestry / fishing etc. |
|  |  |  | 4. Promoting livelihood | 0 | 1 | 2 | 3 | 4 | 5 | A | B | C | *Economy Success Score: Mean value success factors 1-4* | Does the program promote livelihood of people in the area targeted? E.g. are there profitable ecosystem services, jobs created, ecotourism enhancing people’s livelihood? |
|  | | | | | | | | | | | | | | |
|  | **Society** | **Failure Factors** | 1. Conflicts of interest / ownership | -5 | -4 | -3 | -2 | -1 | 0 | A | B | C |  | Are there conflicting interests between stakeholders / landowners and the conservation goals hindering successful implementation of the program? |
|  |  |  | 2. Lack of understanding for needed transformation | -5 | -4 | -3 | -2 | -1 | 0 | A | B | C |  | Do people / stakeholders lack understanding why their behaviors interfere with conservation goals? Is there awareness that a transformation is needed? |
|  |  |  | 3. Bureaucracy and regulations | -5 | -4 | -3 | -2 | -1 | 0 | A | B | C |  | Is there unnecessary bureaucracy and regulations that hinder the program’s successful implementation? Are there regulations that allow sanctions for imperfect measure implementation? |
|  |  |  | 4. Underestimating problem of biodiversity loss | -5 | -4 | -3 | -2 | -1 | 0 | A | B | C | *Society Failure Score: Mean value failure factors 1-4* | Are people underestimating the problem of biodiversity loss leading to limited acceptance of the program? Is biodiversity loss recognized in local communities? |
|  |  | **Success Factors** | 1. Biodiversity awareness | 0 | 1 | 2 | 3 | 4 | 5 | A | B | C |  | Became people more aware of biodiversity and the benefits provided e.g. through ecosystem services? Does this enhance acceptance of the program leading to more successful implementation? |
|  |  |  | 2. Positive outreach, raise interest, grassroot initiatives | 0 | 1 | 2 | 3 | 4 | 5 | A | B | C |  | Are people interested in the program? Are there success stories for a positive outreach promoting grassroot initiatives to support the program? Are NGOs or volunteers engaging in the program? |
|  |  |  | 3. Local acceptance / Collective decision-making | 0 | 1 | 2 | 3 | 4 | 5 | A | B | C |  | Is the program locally accepted? Are local people involved in decision-making and is this promoting long-term success of the program? Is the program respecting values and interests of local stakeholders? |
|  |  |  | 4. Education / Capacity building | 0 | 1 | 2 | 3 | 4 | 5 | A | B | C | *Society Success Score: Mean value success factors 1-4* | Does the program help in education and capacity building for enhancing the long-term success? Does the program provide opportunities for environmental education, e.g. for school or university students? |
|  | | | | | | | | | | | | | | |
|  | **Policy** | **Failure Factors** | 1. Policies damaging biodiversity / Conflicting policies | -5 | -4 | -3 | -2 | -1 | 0 | A | B | C |  | Are there conflicting policies hindering the successful implementation of the program? E.g. Biodiversity Strategy and Renewable Energy Directive. Do other policies harm biodiversity in the area targeted by the program? |
|  |  |  | 2. Lack of science-policy interfaces and counseling | -5 | -4 | -3 | -2 | -1 | 0 | A | B | C |  | Are decision makers insufficiently informed about scientific evidence hindering effective policies? Is there insufficient counseling for decision makers hindering effective program implementation? |
|  |  |  | 3. Lack of awareness | -5 | -4 | -3 | -2 | -1 | 0 | A | B | C |  | Are decision makers not aware enough about conservation issues hindering effectiveness of policies? |
|  |  |  | 4. Lack of action / priorities | -5 | -4 | -3 | -2 | -1 | 0 | A | B | C | *Policy Failure Score: Mean value failure factors 1-4* | Is there a lack of action in program implementation? Are there other political priorities hindering the implementation of the program? |
|  |  | **Success Factors** | 1. Cross-sectoral cooperation and biodiversity mainstreaming | 0 | 1 | 2 | 3 | 4 | 5 | A | B | C |  | Does cross-sectoral cooperation enhance the success of the program? Is biodiversity also considered across sectoral policies (i.e. biodiversity mainstreaming)? |
|  |  |  | 2. Establish fitting conditions through reforms | 0 | 1 | 2 | 3 | 4 | 5 | A | B | C |  | Did political reforms establish fitting conditions enhancing the success of the program? Are measures of the program easy to apply without too many conflicting interests or conflicting polices? |
|  |  |  | 3. Internalize environmental costs | 0 | 1 | 2 | 3 | 4 | 5 | A | B | C |  | Are environmental costs internalized to promote the success of the conservation program? |
|  |  |  | 4. Reliability of funding (ease of access, continuity and sufficiency) | 0 | 1 | 2 | 3 | 4 | 5 | A | B | C | *Policy Success Score: Mean value success factors 1-4* | Is there long-term continuity in funding? E.g. is it easy enough to apply for funding or incentives? Is there a risk to pay back if actions fail? |
|  | | | | | | | | | | | | | | |
|  | | | | | | | | | | | | | | |
|  | **Environment** | **Failure Factors** | 1. Lack of spatial and temporal connectivity | -5 | -4 | -3 | -2 | -1 | 0 | A | B | C |  | Are protected areas / areas of program implementation spatially and temporally connected enough? If not, is this hindering conservation success? |
|  |  |  | 2. Deterioration at other / different places (net loss of natural areas) | -5 | -4 | -3 | -2 | -1 | 0 | A | B | C |  | Is there deterioration of natural areas outside of protected areas? Is there net loss of natural area? Is the area around the area targeted by the program intensively used and has this a negative impact on the protected site? |
|  |  |  | 3. Untargeted conservation | -5 | -4 | -3 | -2 | -1 | 0 | A | B | C |  | Is conservation targeted at specific habitats or species or too untargeted to lead to successful conservation? Is the context of the conservation measures clearly focused on e.g. agricultural landscapes, river ecosystems, marine protected areas etc.? |
|  |  |  | 4. Lack of appropriate monitoring / data | -5 | -4 | -3 | -2 | -1 | 0 | A | B | C | *Environment Failure Score: Mean value failure factors 1-4* | Is the monitoring infrastructure and data present appropriate to implement the conservation program successfully? Is evaluation of success based on existing monitoring data? |
|  |  | **Success Factors** | 1. Understanding ecological context | 0 | 1 | 2 | 3 | 4 | 5 | A | B | C |  | Does the understanding of local ecological context lead to successful implementation of the program? |
|  |  |  | 2. Appropriate management in spatial context | 0 | 1 | 2 | 3 | 4 | 5 | A | B | C |  | Is the local spatial context implemented in the management plans? Are participatory approaches in management successfully implemented? |
|  |  |  | 3. Area size and quality | 0 | 1 | 2 | 3 | 4 | 5 | A | B | C |  | Is the size and quality of the area targeted by the program sufficient? If yes, does this yield in successful conservation? |
|  |  |  | 4. Presence or establishment of local species pools | 0 | 1 | 2 | 3 | 4 | 5 | A | B | C | *Environment Success Score: Mean value success factors 1-4* | Is the local species pool still rich enough (species diversity and genetic diversity) to promote population growth? Is it possible to successfully re-introduce species for successful conservation? Are re-introduced individuals genetically adapted to local conditions? |
|  | | | | | | | | | | | | | | |
| **Evaluated Program:** | | | **Natura 2000** | **WFD** | | | **Rewilding Europe** | | |  |  |  | *Balanced Score: Sum of Success Scores and Failure Scores* |  |

**Supplementary Table S2: Results of the expert evaluations on conservation programs.** The table shows the means and standard deviations of the expert evaluations for each failure (negative) or success factor (positive), as well as the mean scores for each area and the overall balanced score, in total, per program (Natura 2000, Rewilding Europe and the Water Framework Directive [WFD]) and per evaluation round. Below these values, we show the scores of uncertainties in italics, which range from 0 (no uncertainty) to 1 (high uncertainty). Scores for Rewilding Europe marked with asterisks have n=7 due to missing evaluations.

| **Areas and factors** | **Total** | | **Natura 2000** | | **WFD** | | **Rewilding Europe** | |
| --- | --- | --- | --- | --- | --- | --- | --- | --- |
|  | Round 1 | Round 2 | Round 1 | Round 2 | Round 1 | Round 2 | Round 1 | Round 2 |
| ***Overall Balanced Score*** | -3.07 ± 3.79 | -3.28 ± 3.82 | -5.35 ± 2.29 | -5.48 ± 2.60 | -3.10 ± 3.11 | -3.63 ± 2.49 | 0.89 ± 3.76 | 0.96 ± 3.95 |
| *Economy* | -1.23 ± 1.24 | -1.47 ± 1.03 | -1.71 ± 1.09 | -2.02 ± 0.73 | -1.35 ± 1.13 | -1.55 ± 0.86 | -0.38 ± 1.26 | -0.53 ± 1.04 |
| F1. Subsidies damaging biodiversity | -4.10 ± 0.88  *0.17* | -4.33 ± 0.61  *0.13* | -4.08 ± 1.00  *0.13* | -4.42 ± 0.67  *0.08* | -4.00 ± 0.94  *0.30* | -4.20 ± 0.63  *0.30* | -4.25 ± 0.71  *0.06* | -4.38 ± 0.52  *0.00* |
| F2. Economic interests competing with conservation | -3.90 ± 1.03  *0.22* | -4.07 ± 0.87  *0.20* | -4.17 ± 0.83  *0.13* | -4.42 ± 0.67  *0.13* | -4.10 ± 0.74  *0.25* | -4.10 ± 0.74  *0.25* | -3.25 ± 1.39  *0.31* | -3.50 ± 1.07  *0.25* |
| F3. Lack of funding | -3.63 ± 1.61  *0.15* | -3.83 ± 1.32  *0.13* | -4.33 ± 1.15  *0.04* | -4.67 ± 0.49  *0.04* | -3.60 ± 1.90  *0.20* | -3.80 ± 1.40  *0.20* | -2.63 ± 1.41  *0.25* | -2.63 ± 1.19  *0.19* |
| F4. Unsustainable use of resources | -2.77 ± 1.41  *0.47* | -3.00 ± 1.08  *0.42* | -3.42 ± 1.00  *0.38* | -3.50 ± 0.90  *0.33* | -2.40 ± 1.51  *0.50* | -2.70 ± 1.06  *0.50* | -2.25 ± 1.58  *0.56* | -2.63 ± 1.19  *0.44* |
| S1. Incentives and subsidies | 2.77 ± 1.17  *0.25* | 2.70 ± 1.12  *0.27* | 2.92 ± 1.00  *0.29* | 2.83 ± 0.94  *0.29* | 2.80 ± 1.14  *0.30* | 2.70 ± 1.06  *0.30* | 2.50 ± 1.51  *0.13* | 2.50 ± 1.51  *0.19* |
| S2. Strict production standards / supply regulations | 1.70 ± 1.42  *0.43* | 1.80 ± 1.24  *0.42* | 2.08 ± 1.16  *0.38* | 2.17 ± 1.03  *0.38* | 1.20 ± 1.40  *0.45* | 1.30 ± 1.25  *0.45* | 1.75 ± 1.75  *0.50* | 1.88 ± 1.46  *0.44* |
| S3. Certification of production | 2.07 ± 1.39  *0.47* | 1.90 ± 1.16  *0.43* | 2.08 ± 1.56  *0.33* | 1.92 ± 1.31  *0.25* | 2.00 ± 1.49  *0.65* | 1.70 ± 1.06  *0.65* | 2.13 ± 1.13  *0.44* | 2.13 ± 1.13  *0.38* |
| S4. Promoting livelihood | 2.93 ± 1.53  *0.30* | 2.93 ± 1.39  *0.28* | 2.08 ± 1.16  *0.46* | 1.92 ± 1.08  *0.38* | 2.70 ± 1.49  *0.35* | 2.90 ± 0.88  *0.40* | 4.50 ± 0.76  *0.00* | 4.50 ± 0.76  *0.00* |
| *Society* | -0.62 ± 1.24 | -0.59 ± 1.17 | -1.21 ± 1.13 | -1.13 ± 0.96 | -0.90 ± 0.50 | -0.95 ± 0.51 | 0.63 ± 1.26 | 0.66 ± 1.21 |
| F1. Conflicts of interest / ownership | -4.13 ± 1.01  *0.10* | -4.13 ± 0.97  *0.07* | -4.50 ± 0.67  *0.04* | -4.58 ± 0.51  *0.04* | -4.40 ± 0.52  *0.10* | -4.30 ± 0.48  *0.10* | -3.25 ± 1.39  *0.19* | -3.25 ± 1.39  *0.06* |
| F2. Lack of understanding for needed transformation | -3.00 ± 1.17  *0.35* | -3.00 ± 0.95  *0.35* | -2.83 ± 1.11  *0.50* | -2.83 ± 0.58  *0.50* | -3.30 ± 1.16  *0.30* | -3.50 ± 0.85  *0.30* | -2.88 ± 1.36  *0.19* | -2.63 ± 1.30  *0.19* |

| **Areas and factors** | **Total** | | **Natura 2000** | | **WFD** | | **Rewilding Europe** | |
| --- | --- | --- | --- | --- | --- | --- | --- | --- |
|  | Round 1 | Round 2 | Round 1 | Round 2 | Round 1 | Round 2 | Round 1 | Round 2 |

| F3. Bureaucracy and regulations | -3.23 ± 1.30  *0.28* | -3.33 ± 0.99  *0.25* | -3.50 ± 1.38  *0.25* | -3.50 ± 1.24  *0.17* | -2.70 ± 1.25  *0.30* | -3.00 ± 0.67  *0.30* | -3.50 ± 1.20  *0.31* | -3.50 ± 0.93  *0.31* |
| --- | --- | --- | --- | --- | --- | --- | --- | --- |
| F4. Underestimating problem of biodiversity loss | -3.10 ± 1.27  *0.35* | -3.13 ± 1.01  *0.33* | -3.25 ± 1.42  *0.33* | -3.17 ± 0.94  *0.33* | -3.40 ± 1.17  *0.30* | -3.50 ± 0.97  *0.30* | -2.50 ± 1.07  *0.44* | -2.63 ± 1.06  *0.38* |
| S1. Biodiversity awareness | 2.63 ± 1.27  *0.28* | 2.67 ± 1.15  *0.30* | 2.17 ± 1.11  *0.29* | 2.08 ± 0.90  *0.33* | 2.80 ± 1.23  *0.35* | 2.90 ± 1.10  *0.35* | 3.13 ± 1.46  *0.19* | 3.25 ± 1.28  *0.19* |
| S2. Positive outreach, raise interest, grassroot initiatives | 3.23 ± 1.25  *0.27* | 3.20 ± 1.16  *0.28* | 2.83 ± 1.19  *0.38* | 2.92 ± 1.08  *0.38* | 3.00 ± 1.33  *0.25* | 2.80 ± 1.14  *0.30* | 4.13 ± 0.83  *0.13* | 4.13 ± 0.83  *0.13* |
| S3. Local acceptance / Collective decision-making | 2.90 ± 1.12  *0.25* | 3.00 ± 0.98  *0.25* | 2.42 ± 0.90  *0.38* | 2.58 ± 0.67  *0.38* | 2.40 ± 0.70  *0.25* | 2.50 ± 0.53  *0.25* | 4.25 ± 0.71  *0.06* | 4.25 ± 0.71  *0.06* |
| S4. Education / Capacity building | 2.23 ± 1.41  *0.28* | 2.33 ± 1.27  *0.32* | 1.83 ± 1.19  *0.33* | 1.92 ± 1.24  *0.38* | 2.00 ± 1.25  *0.25* | 2.30 ± 0.95  *0.25* | 3.13 ± 1.64  *0.25* | 3.00 ± 1.51  *0.31* |
| *Policy* | -1.08 ± 1.26 | -1.12 ± 1.22 | -1.71 ± 0.86 | -1.58 ± 0.89 | -0.85 ± 1.14 | -1.00 ± 1.20 | -0.44 ± 1.58 | -0.56 ± 1.53 |
| F1. Policies damaging biodiversity / Conflicting policies | -3.87 ± 1.04  *0.25* | -4.00 ± 0.83  *0.22* | -4.00 ± 0.74  *0.25* | -4.17 ± 0.58  *0.21* | -3.70 ± 1.49  *0.20* | -3.80 ± 1.14  *0.25* | -3.88 ± 0.83  *0.31* | -4.00 ± 0.76  *0.19* |
| F2. Lack of science-policy interfaces and counselling | -3.07 ± 1.17  *0.45* | -3.17 ± 1.12  *0.40* | -3.58 ± 0.90  *0.42* | -3.67 ± 0.89  *0.33* | -2.60 ± 1.17  *0.50* | -2.70 ± 1.16  *0.50* | -2.88 ± 1.36  *0.44* | -3.00 ± 1.20  *0.38* |
| F3. Lack of awareness | -2.80 ± 1.37  *0.45* | -2.93 ± 1.23  *0.43* | -3.17 ± 1.34  *0.38* | -3.08 ± 1.24  *0.33* | -2.30 ± 1.16  *0.55* | -2.70 ± 1.06  *0.60* | -2.88 ± 1.64  *0.44* | -3.00 ± 1.51  *0.38* |
| F4. Lack of action / priorities | -3.43 ± 1.59  *0.28* | -3.50 ± 1.36  *0.27* | -4.00 ± 1.21  *0.33* | -3.92 ± 1.08  *0.25* | -3.90 ± 1.29  *0.20* | -4.00 ± 0.67  *0.25* | -2.00 ± 1.69  *0.31* | -2.25 ± 1.67  *0.31* |
| S1. Cross-sectoral cooperation and biodiversity mainstreaming | 3.07 ± 1.53  *0.30* | 3.17 ± 1.26  *0.28* | 2.92 ± 1.56  *0.29* | 3.00 ± 1.41  *0.29* | 3.50 ± 1.51  *0.40* | 3.60 ± 1.17  *0.40* | 2.75 ± 1.58  *0.19* | 2.88 ± 1.13  *0.13* |
| S2. Establish fitting conditions through reforms | 2.00 ± 1.26  *0.45* | 2.07 ± 1.28  *0.40* | 1.92 ± 1.31  *0.42* | 2.08 ± 1.38  *0.38* | 1.60 ± 1.07  *0.45* | 1.60 ± 1.07  *0.45* | 2.63 ± 1.30  *0.50* | 2.63 ± 1.30  *0.38* |
| S3. Internalize environmental costs | 1.40 ± 1.16  *0.47* | 1.33 ± 1.06  *0.47* | 1.08 ± 0.90  *0.46* | 1.08 ± 0.90  *0.50* | 1.50 ± 1.08  *0.45* | 1.30 ± 0.67  *0.45* | 1.75 ± 1.58  *0.50* | 1.75 ± 1.58  *0.44* |
| S4. Reliability of funding (ease of access, continuity and sufficiency) | 2.37 ± 1.33  *0.35* | 2.57 ± 1.19  *0.33* | 2.00 ± 1.35  *0.42* | 2.33 ± 1.30  *0.38* | 2.50 ± 1.35  *0.40* | 2.70 ± 1.06  *0.40* | 2.75 ± 1.28  *0.19* | 2.75 ± 1.28  *0.19* |

| **Areas and factors** | **Total** | | **Natura 2000** | | **WFD** | | **Rewilding Europe** | |
| --- | --- | --- | --- | --- | --- | --- | --- | --- |
|  | Round 1 | Round 2 | Round 1 | Round 2 | Round 1 | Round 2 | Round 1 | Round 2 |

| *Environment* | 0.03 ± 1.39 | 0.03 ± 1.40 | -0.73 ± 1.32 | -0.75 ± 1.29 | 0.00 ± 0.95 | -0.13 ± 0.65 | 1.39 ± 1.04^*^ | 1.57 ± 1.21^*^ |
| --- | --- | --- | --- | --- | --- | --- | --- | --- |
| F1. Lack of spatial and temporal connectivity | -3.50 ± 1.20  *0.18* | -3.53 ± 0.97  *0.12* | -3.58 ± 1.08  *0.21* | -3.67 ± 1.07  *0.08* | -3.50 ± 1.58  *0.20* | -3.50 ± 0.97  *0.20* | -3.38 ± 0.92  *0.13* | -3.38 ± 0.92  *0.06* |
| F2. Deterioration at other / different places (net loss of natural areas) | -3.69 ± 1.11  *0.28* | -3.76 ± 1.09  *0.24* | -4.17 ± 0.83  *0.21* | -4.33 ± 0.65  *0.17* | -3.90 ± 0.99  *0.25* | -3.90 ± 0.99  *0.25* | -2.57 ± 0.98^*^  *0.43* | -2.57 ± 0.98^*^  *0.36* |
| F3. Untargeted conservation | -2.10 ± 1.56  *0.27* | -2.14 ± 1.30  *0.28* | -2.92 ± 1.24  *0.29* | -2.83 ± 1.11  *0.33* | -1.20 ± 1.40  *0.25* | -1.40 ± 1.07  *0.25* | -2.00 ± 1.69  *0.25* | -2.00 ± 1.41^*^  *0.21* |
| F4. Lack of appropriate monitoring / data | -2.63 ± 1.43  *0.17* | -2.43 ± 1.30  *0.13* | -3.25 ± 1.22  *0.17* | -3.00 ± 0.95  *0.13* | -1.90 ± 1.37  *0.15* | -1.80 ± 1.03  *0.15* | -2.63 ± 1.51  *0.19* | -2.38 ± 1.77  *0.13* |
| S1. Understanding ecological context | 3.30 ± 1.21  *0.30* | 3.27 ± 1.11  *0.30* | 3.17 ± 1.27  *0.33* | 3.08 ± 1.00  *0.33* | 3.20 ± 1.14  *0.35* | 3.20 ± 1.14  *0.35* | 3.63 ± 1.30  *0.19* | 3.63 ± 1.30  *0.19* |
| S2. Appropriate management in spatial context | 2.87 ± 1.25  *0.37* | 2.87 ± 1.17  *0.38* | 2.42 ± 1.38  *0.38* | 2.42 ± 1.16  *0.42* | 2.50 ± 0.85  *0.40* | 2.50 ± 0.85  *0.40* | 4.00 ± 0.76  *0.31* | 4.00 ± 0.76  *0.31* |
| S3. Area size and quality | 2.87 ± 1.28  *0.32* | 2.73 ± 1.17  *0.28* | 2.42 ± 1.16  *0.29* | 2.25 ± 0.97  *0.25* | 2.60 ± 1.43  *0.35* | 2.40 ± 1.17  *0.40* | 3.88 ± 0.64  *0.31* | 3.88 ± 0.64  *0.19* |
| S4. Presence or establishment of local species pools | 3.10 ± 1.32  *0.31* | 3.03 ± 1.35  *0.29* | 3.00 ± 1.13  *0.46* | 2.92 ± 1.08  *0.42* | 2.20 ± 1.03  *0.25* | 2.00 ± 0.82  *0.25* | 4.57 ± 0.53^*^  *0.14* | 4.71 ± 0.49^*^  *0.14* |

**Supplementary Table S3: Summary of the GLMM results.** The table shows mixed model analyses of variance of the effects of conservation programs (Natura 2000 vs. Water Framework Directive vs. Rewilding Europe), area (economy vs. society vs. policy vs. environment) and type (success vs. failure) of a factor on expert scores during the survey.

| **Fixed factors (and their interactions)** | **df** | **χ²** | **p** |
| --- | --- | --- | --- |
| Conservation program | 2 | 24.06 | < 0.001 |
| Area | 3 | 59.26 | < 0.001 |
| Type | 1 | 183.88 | < 0.001 |
| Program x Area | 6 | 8.38 | 0.211 |
| Program x Type | 2 | 8.14 | 0.017 |
| Area x Type | 3 | 7.71 | 0.052 |
| Program x Area x Type | 6 | 15.84 | 0.015 |


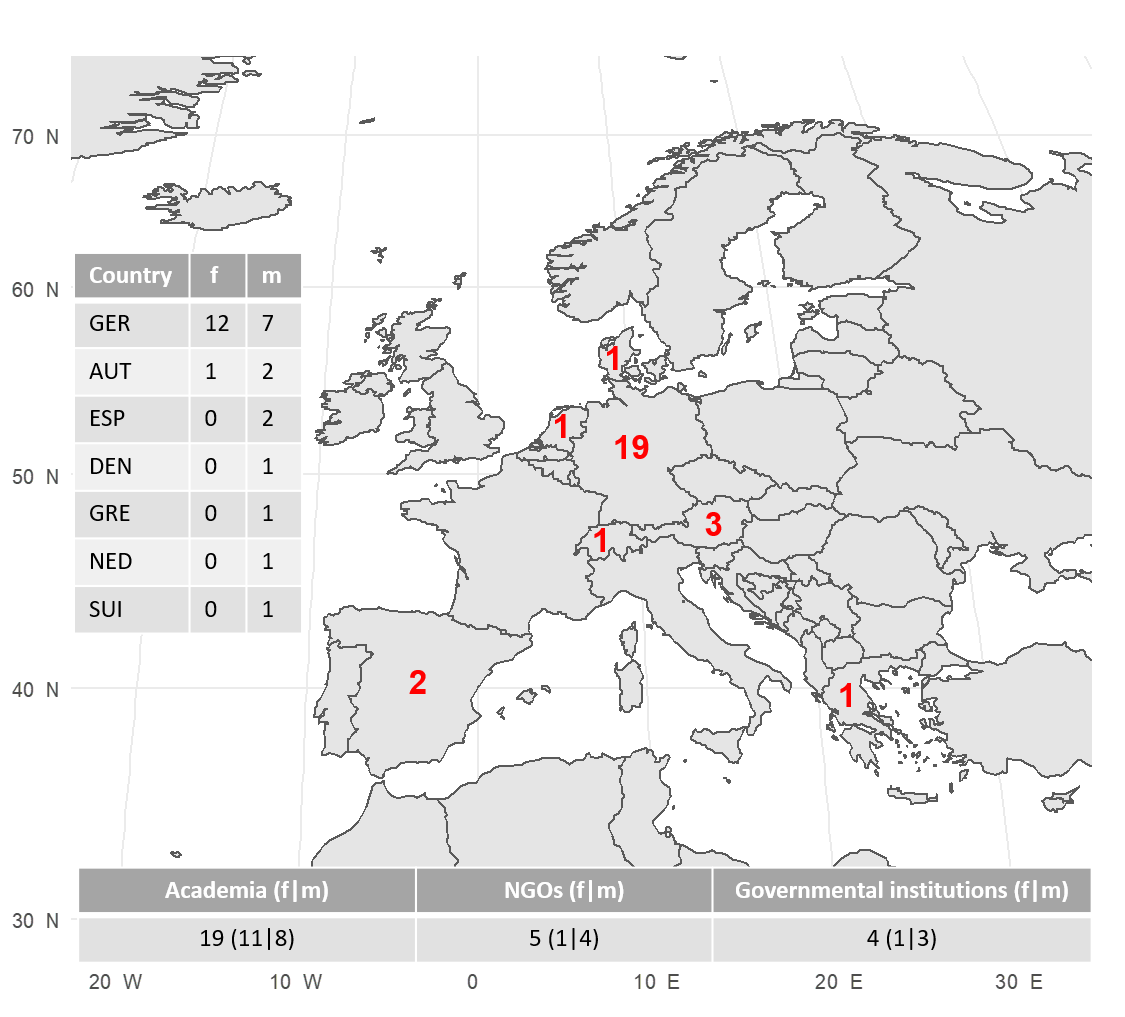


**Supplementary Figure S1: Geographic and demographic information on the experts that filled in the balanced scorecards.** The map shows the number of experts per country (in red), the table on the left shows the gender-balance per country and the table on the bottom shows the professional background of the experts per gender.


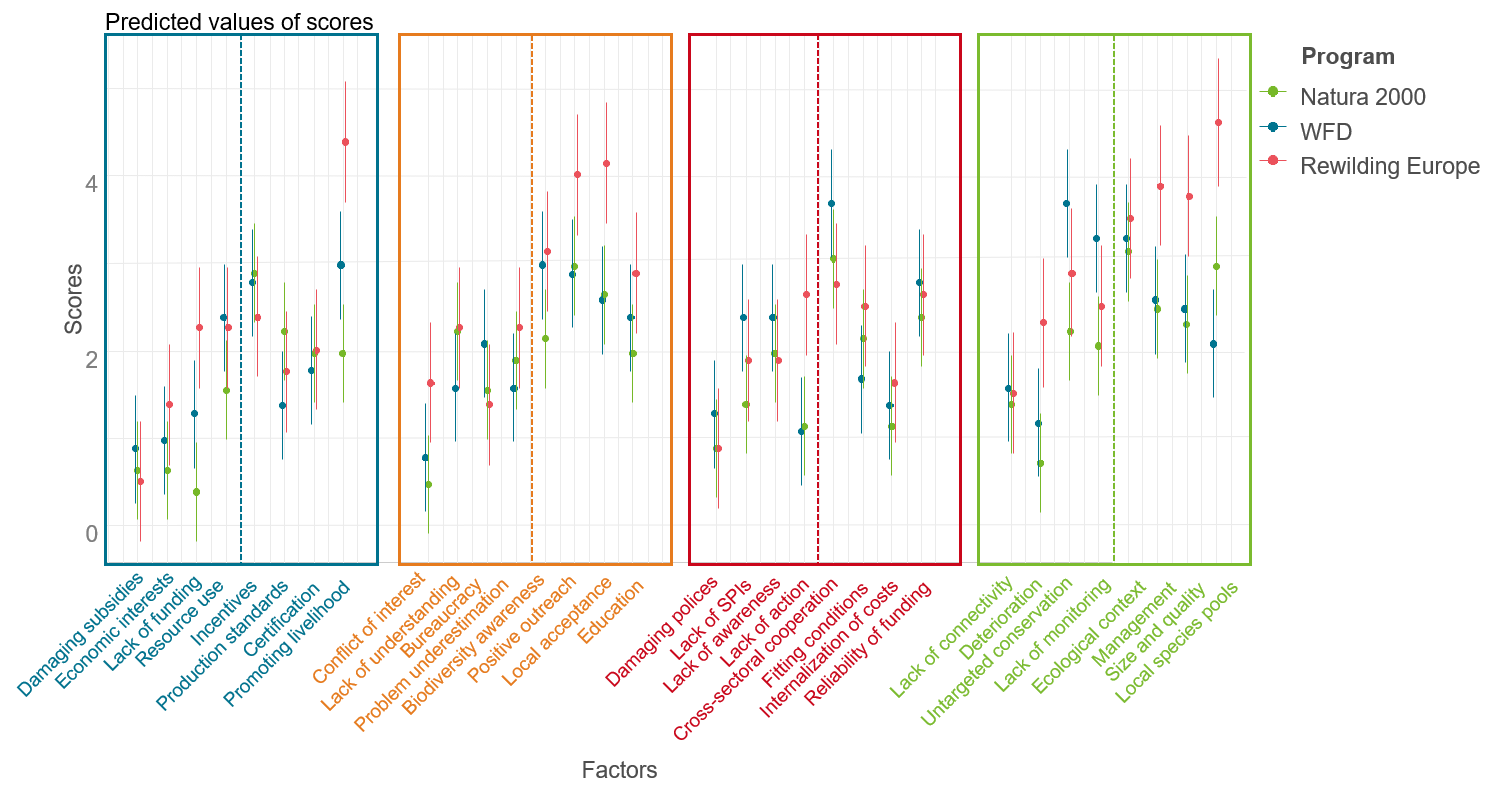


**Supplementary Figure S2: Predicted values of failure and success factors from the linear mixed-effects model.** The plots show the mean scores per factor with 95% confidence intervals for each of the programs separately. Note that values of failure factors (first four of each area) were transformed by adding +5, i.e. low values for failure factors refer to strong impact on failure, while high impact on success is indicated by high scores.
